# Contrahemispheric Cortex Predicts Survival and Molecular Markers in Patients With Unilateral High-Grade Gliomas

**DOI:** 10.3389/fonc.2020.00953

**Published:** 2020-07-23

**Authors:** Taoyang Yuan, Jianyou Ying, Zhentao Zuo, Lu Jin, Songbai Gui, Zhixian Gao, Guilin Li, Rui Wang, Yazhuo Zhang, Chuzhong Li

**Affiliations:** ^1^Beijing Neurosurgical Institute, Capital Medical University, Beijing, China; ^2^State Key Laboratory of Brain and Cognitive Science, Institute of Biophysics, Chinese Academy of Sciences, Beijing, China; ^3^Department of Neurosurgery, Beijing Tiantan Hospital, Capital Medical University, Beijing, China; ^4^China National Clinical Research Center for Neurological Diseases, Beijing, China; ^5^Beijing Institute for Brain Disorders Brain Tumor Center, Beijing, China

**Keywords:** high-grade gliomas, brain structure, MRI, molecular markers, overall survival

## Abstract

**Background:** Malignant high-grade gliomas are characterized by infiltration and destruction of surrounding brain tissue. Alterations in the contrahemispheric brain structure and their roles that may offer prognostically valuable information have not been investigated in high-grade gliomas.

**Methods:** In total, 153 patients with unilateral glioma (low-grade, *n* = 77; high-grade, *n* = 76) and 115 healthy controls (HCs) were recruited and scanned with 3-D T1 imaging. The gray matter (GM), white matter (WM), and cerebrospinal fluid (CSF) volume in the contrahemisphere were examined. Partial correlation, logistic regression, and multivariate Cox's regression analyses were performed.

**Results:** The contrahemispheric GM volume (CHGMV) in the high-grade glioma patients was significantly decreased compared to that in the HCs/low-grade gliomas (one-way ANOVA, Bonferroni corrected, *p* < 0.05). The CHGMV is significantly correlated with the WHO grade (*r* = −0.22, *p* < 0.05) and contrast-enhanced volume (*r* = −0.33, *p* < 0.01). In the high-grade gliomas, the binary logistic regression revealed that the CHGMV can independently predict isocitrate dehydrogenase 1 (IDH1) and P53 mutations. The survival curves revealed that the patients with a low CHGMV had a shorter overall survival (OS) than the patients with a high CHGMV (*p* = 0.001). The multivariate Cox's regression analysis showed that a low CHGMV can independently predict unfavorable OS with a hazard ratio of 2.883 (*p* = 0.035).

**Conclusions:** Volume of the contrahemispheric cortex can be potentially used in clinical practice as an imaging biomarker to predict survival and molecular markers in patients with unilateral high-grade gliomas.

## Introduction

Malignant high-grade gliomas (WHO grade III and IV) are the most common primary brain cancers in adults and have a dismal prognosis despite the use of microsurgical resection, followed by chemotherapy and radiotherapy (1–3). High-grade gliomas are characterized by diffuse infiltration and the destruction of surrounding brain tissue, even invasive remote brain regions along white matter (WM) tracts (4). The clinical management of this patient population can be very challenging, especially prognostic evaluation.

The imaging, pathologic, and molecular features of malignant glioma provide opportunities for subclassification, prognostication, and the development of targeted therapies (3, 5–8). Conventional magnetic resonance imaging (MRI) plays an important role in neuro-oncology for the initial diagnosis and assessment of treatment response (9). The development of a quantitative assessment using advanced magnetic resonance techniques, including diffusion, perfusion, spectroscopy, tractography, and structural and functional MRI, could enable accurate, non-invasive investigation of the brain structure and function alterations and aid in designing treatment plans, assessing tumor malignancy and molecular status, and predicting prognosis in patients with glioma (7, 10–12). In radiomics, the MRI characteristics of brain gliomas, such as the volumes of contrast enhancement, edema, and necrosis; sharpness of lesion boundaries; boundary shapes; and the ratio of the T2/FLAIR hyper-intense volume to the volume of contrast enhancement and necrosis, have been reported to predict the tumor grade, molecular markers representing genes (IDH1, p53, ATRX, and MGMT), the efficacy of chemoradiotherapy, and glioma patient survival (7, 13–17). An integrative analysis of various MR imaging data extracted from tumors using modern machine learning technology could provide more accurate and reproducible predictions (18). Interestingly, large-scale brain functional networks can also be used to predict overall (OS) survival in high-grade glioma patients (19).

The poor survival rates are considered to be caused by the aggressive nature of high-grade gliomas. In high-grade gliomas, immunohistochemical data based on the isocitrate–dehydrogenase (IDH) mutation status in a previous study revealed that glial tumor cells migrate into multiple brain regions, including the contralesional hemisphere, and redefined glioma as a whole-brain disease rather than a focal brain disease (20). Recently, Jütten et al. (21) found microstructural alterations in whole-brain normal-appearing WM in patients with glioma using diffusion tensor imaging (DTI), which was associated with cognitive dysfunction. Considering these findings, the clinical management of high-grade glioma patients, including the assessment of the tumor molecular status, prediction of prognosis, and disease monitoring strategies, focusing only on the focal tumor, seems to be insufficient. To the best of our knowledge, no study has investigated the effects of high-grade gliomas on whole-brain gray matter (GM) *in vivo* or whether the change in GM is correlated with survival and genomic signatures.

In the current study, we examine changes in the GM, WM, and cerebrospinal fluid (CSF) volume within the hemisphere without the tumor burden in low- and high-grade glioma patients and healthy controls (HCs). Considering that the extent of the tumor burden on contrahemispheric cortex might offer prognostically valuable information, we further explored the possibility of using the contrahemispheric brain structure extracted from T1 images to predict survival and molecular markers in patients with high-grade gliomas.

## Methods

### Participants

A total of 153 patients with a pathologically confirmed glioma (WHO grades II–IV) affecting the unilateral hemisphere were enrolled in our study at Beijing Tiantan Hospital from September 2016 to December 2017. The clinical variables including age, sex, preoperative Karnofsky performance score (KPS), tumor types, tumor location, and pathological information, were obtained. Among these subjects, 40 patients with left low-grade glioma (LLGG group), 36 patients with left high-grade glioma (LHGG group), 37 patients with right low-grade glioma (RLGG group), and 40 patients with right high-grade glioma (RHGG group) were included. The inclusion criteria for the patient group were an age within the range of 16–70 years, a tumor located in a unilateral cerebral hemisphere above the tentorium cerebellum, and histologically proven glioma. The exclusion criteria were as follows: a history of stroke, cerebral trauma, brain surgery, recurrent glioma, brain radiotherapy, chemotherapy treatment, shift of the midline due to tumor space occupation, bilateral extension of the lesion, an inability to complete MRI examinations, or preprocessing issues (i.e., head motion).

The HCs included 115 neurologically intact participants. Individuals with a history of neurodegenerative diseases, neurodevelopmental or psychiatric diseases, substance use disorders related to alcohol or heroin, an inability to complete MRI examinations, or preprocessing issues (i.e., head motion) were excluded.

### Image Acquisition

All subjects were scanned using a 3.0 Tesla Siemens scanner with a standard head coil. The 3-D T1-weighted sagittal anatomical image was acquired (192 slices, slice thickness/gap = 1/0.5 mm, repetition time = 2,530 ms, echo time = 2.55 ms, acquisition matrix = 512 × 512, flip angle = 12°, FOV = 256 × 256 mm and an in-plane resolution of 0.7 × 0.7 mm). The T2 image parameters were as follows: repetition time = 5,000 ms, echo time = 105 ms, flip angle = 150°, 33 slices, field of view = 199 × 220 mm^2^, voxel size = 0.49 × 0.49 × 3.9 mm^3^, and matrix = 406 × 448. All patients underwent the MRI scan using the same protocol in the same machine in the same department.

The postcontrast T1-weighted images were acquired using a 3.0 Tesla Siemens or GE scanner after an injection of gadopentetate dimeglumine (Ga-DTPA injection, Beilu Pharma, Beijing, China) at a dose of 0.1 mmol/kg using an echo time (TE) of 15 ms, a repetition time (TR) of 450 ms, and a slice thickness of 5 mm.

### Tumor Masking

**Figure 1:**
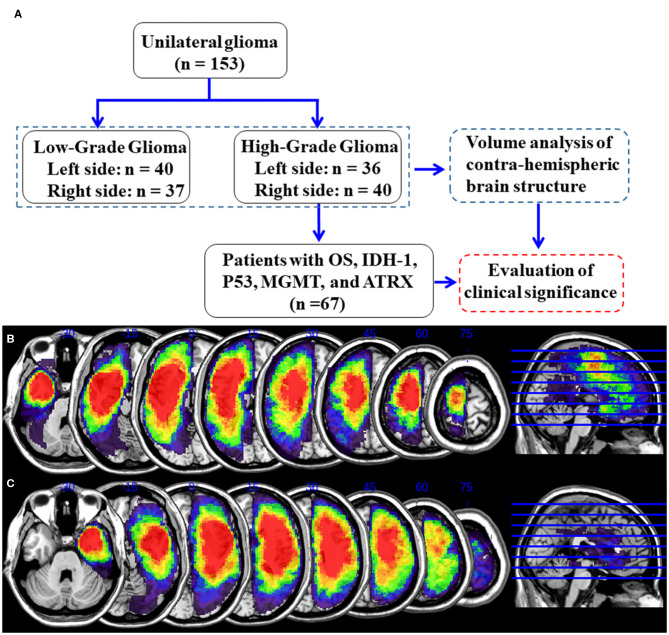
Detailed flow chart of the analysis and glioma overlap map. **(A)** Flowchart of analysis. **(B)** Left hemisphere glioma overlap map. **(C)** Right hemisphere glioma overlap map.

The tumor masking was traced with MRIcron software (https://www.mccauslandcenter.sc.edu/crnl/tools) for each patient on a T2 image in the native space. To define the anatomical location of the tumor in each patient, the T2 image and tumor mask of each patient were registered to the Montreal Neurological Institute template using the standard non-linear spatial normalization algorithm provided by SPM12. Finally, all tumor masks were overlapped using the Ch2bet template. The tumor overlap maps of the left hemisphere gliomas and right hemisphere gliomas are displayed in Figures 1B,C. The lesion volumes were assessed using MRIcron based on the lesion drawing.

### Contrast Enhanced Volume

The contrast enhancement of the tumor was assessed by two neurosurgeons who were blinded to our study. The contrast enhanced volume of the gliomas, including areas of contrast enhancement and areas of central necrosis, was measured using postcontrast T1-weighted images (22).

### Immunohistochemistry for the Detection of IDH1-R132H, MGMT, ATRX, and P53

The IDH1 status, P53 status, O-6-methylguanineDNA methyltransferase (MGMT) promoter methylation, and alpha-thalassemia/mental retardation, X-linked (ATRX) expression in the current study were routinely evaluated by experienced pathologists using typical tumor samples collected from the glioma patients. Immunostaining was performed in accordance with the manufacturer's protocol. ATRX, P53, MGMT, and IDH1 immunostains were performed on an automated immunohistochemistry system (BenchMark ULTRA, Ventana Medical Systems, Strasbourg, France). Anti-human IDH1-R132H, anti-human ATRX, anti-human MGMT, and anti-human P53 antibodies were purchased from Zhongshan Golden Bridge Biotechnology (Beijing, China). For IDH1-R312H staining, a strong cytoplasmic immunoreaction product was scored as positive, and weak, diffuse staining and macrophage staining were scored as negative (23). The loss of nuclear ATRX expression was scored as specific, and if the tumor cell nuclei were unstained while the nuclei of non-neoplastic cells, such as endothelia, microglia, lymphocytes, and reactive astrocytes, were stained, the staining was considered strongly positive (24). The expression of P53 was considered positive when the proportion of positive cells was >10% (25). Two pathologists blinded to the clinical data scored the degree of staining.

### Image Preprocessing

The structural image preprocessing was conducted with Statistical Parametric Mapping (SPM12, Wellcome Trust Center for NeuroImaging, University College, London, UK) running in the MATLAB environment (release 2015a, MathWorks, Inc, Natick, MA). First, the structural images were manually reoriented and shifted to define the anterior commissure as the origin (mm coordinate 0,0,0). Then, the 3-D T1-weighted images were segmented with the segment module implemented in SPM12 into GM, WM, and CSF in the native space. This function uses the unified segmentation algorithm, which combines image registration, tissue classification, and bias correction. The GM, WM, and CSF maps were normalized with modulation. Finally, the amount of smoothing was set at 8-mm Gaussian full width at half-maximum.

The GM, WM, and CSF volumetric analyses were restricted to the tumor-free contralateral hemisphere. Similarly, the GM, WM, and CSF volumetric analyses in healthy controls were also restricted to a unilateral hemisphere. To extract the volumetric data of the contralateral hemisphere, a unilateral hemisphere mask was generated using the WFU_PickAtlas toolbox in SPM12 based on Talairach Daemon labels (26). The unilateral hemisphere mask was co-registered to a standard template and re-sliced to 1.5 × 1.5 × 1.5 mm^3^ voxel size in the Montreal Neurological Institute space. We obtained the volume of the contrahemispheric GM, WM, and CSF in glioma patients and the volume of the left and right unilateral hemispheric GM, WM, and CSF in the HCs from modulated normalized GM, WM, and CSF data sets, respectively, using the MATLAB script get_totals provided by Ridgway (www0.cs.ucl.ac.uk/staff/g.ridgway/vbm/get_totals.m). The total intracranial volume (TIV) of the unilateral hemisphere was calculated as the sum of the unilateral hemispheric primary GM volume, WM volume, and CSF volume. To correct for variation in the subjects' head sizes, the contralesional hemisphere GM volume (CHGMV), WM volume (CHWMV), and CSF volume (CHCSFV) were calculated by dividing the individual subjects' unilateral hemispheric primary GM volume, WM volume, and CSF volume by each subject's respective unilateral hemispheric TIV. Finally, the contra-lesional volumes in the patients were compared with those on the same side in the HCs.

### Statistical Analyses

A one-way ANOVA with Bonferroni correction was used to assess the difference in CHGMV, CHWMV, and CHCSFV among the patient groups and HCs. The correlations of volumes with glioma grade and contrast-enhanced volume were assessed by partial correlation analysis after correcting for age. The χ^2^-test was used to assess the difference in sex, tumor types, and tumor locations among the groups. A binary logistic regression model was used to investigate predictors of IDH1/P53 mutation and ATRX loss and is presented using odds ratios (ORs) with its 95% confidence intervals (CIs). Kaplan–Meier curves and log-rank tests were performed for survival analysis in high-grade glioma patients. Univariate analyses of the different variables were performed with 95% CIs followed by Cox multivariate logistic regression analyses and hazard ratio (HR) calculations. These analyses were carried out using Statistical Package for the Social Science (SPSS) package (SPSS Inc., version 25.0, Chicago, IL), with a two-tailed significance level of *p* < 0.05. Effect sizes (ES) and their respective 95% CIs in the significant group differences were calculated using Cohen's *d* (27).

## Results

### Demographic and Clinical Factors

One hundred fifty-three patients with unilateral glioma (LLGG group: *n* = 40, 18 females, average age = 39.1 ± 10.8 years; LHGG group: *n* = 36, 16 females, average age = 44.8 ± 11.4 years; RLGG group: *n* = 37, 15 females, average age = 39.8 ± 8.9 years; RHGG group: *n* = 40, 20 females, average age = 45.9 ± 13.3 years) and 115 HCs (60 females, average age = 42.8 ± 11.1 years) were included. All patients accepted surgical treatment, and the postoperative pathological diagnosis was glioma. Not all patients collected the entire pathological index (IDH1, ATRX, MGMT, and P53). No significant differences in age, sex, and hemispheric volumes were observed among the groups. The demographic and clinical features are shown in Table 1. In the high-grade glioma group (*n* = 76), all patients received standard-of-care treatment, including surgical resection followed by radiation therapy and concurrent and adjuvant temozolomide except for six patients who were lost to follow-up. The OS, which was defined as the time between the initial surgical treatment and death due to the tumor or the last follow-up was obtained (*n* = 70). In total, 67 patients with high-grade glioma had complete OS and pathological index (IDH1, ATRX, MGMT, and P53) data. Predictive tumor molecular markers and OS were analyzed in these high-grade glioma patients. Among 67 high-grade glioma patients, the median OS was 23.6 months (range, 6.0–30.0 months). A detailed flow chart of the analysis is shown in Figure 1A. The detailed information about tumor types and locations in the glioma patients is shown in Supplementary Table 1. The results showed no significant differences in tumor types and locations among the subgroups.

**Table 1:** Demographic and clinical characteristics among four patient groups and healthy controls (HCs).

**Variable**	**LLGG group**	**LHGG group**	**RLGG group**	**RHGG group**	**HCs**	***P*-value**
Numbers	40	36	37	40	115	NA
Age, year	39.1 ± 10.8	44.8 ± 11.4	39.8 ± 8.9	45.9 ± 13.3	42.8 ± 11.1	*P* = 0.06^a^
Sex ratio, F/M, *n*	18/22	16/20	15/22	20/20	60/55	*P* = 0.73^b^
Handedness, R/A/L, *n*	38/1/1	34/1/1	35/2/0	38/1/1	110/2/3	NA
Tumor grade, I/II, III/IV, *n*	0/40	23/13	0/37	16/24	NA	NA
Lesion volume, cm^3^	50.6 ± 38.3	81.0 ± 42.3	47.1 ± 37.1	97.6 ± 60.6	NA	NA
Contrast enhanced volume, cm^3^	2.48 ± 14.56	20.12 ± 25.27	0.48 ± 1.61	33.89 ± 34.51	NA	NA
Right hemispheric volume, cm^3^	593.0 ± 55.8	587.9 ± 54.3	NA	NA	586.5 ± 61.8	*P* = 0.84^a^
Left hemispheric volume, cm^3^	NA	NA	584.4 ± 54.8	584.4 ± 53.8	580.1 ± 60.6	*P* = 0.88^a^
KPS	86.0 ± 7.1	79.4 ± 14.7	85.4 ± 9.0	77.8 ± 17.5	100	*P* < 0.01^a^
**Pathological index**						
IDH1_M_/ IDH1_WT_, *n*	31/5	19/13	30/6	19/17	NA	NA
ATRX_Exp_/ATRX_Loss_, *n*	12/22	14/19	13/22	20/17	NA	NA
P53_M_/P53_WT_, *n*	19/15	20/12	10/26	18/18	NA	NA
MGMT promoter methylation (U/M), *n*	7/24	2/32	3/31	6/32	NA	NA

a *represent one-way ANOVA*.

b *represent χ^2^-test*.

*The mean values and standard deviation are given for age, lesion volume, contrast-enhanced volume, hemispheric volume, and KPS. Abbreviations: KPS, Karnofsky performance score; IDH1_M_, IDH1 mutation; IDH1_WT_, IDH1 wild type; ATRX_Exp_, ATRX expression; ATRX_Loss_, ATRX loss; P53_M_, P53 mutation; P53_WT_, P53 wild type. U, unmethylated; M, methylated*.

### Brain Structure Alterations in the Patients With High-Grade Glioma

**Figure 2:**
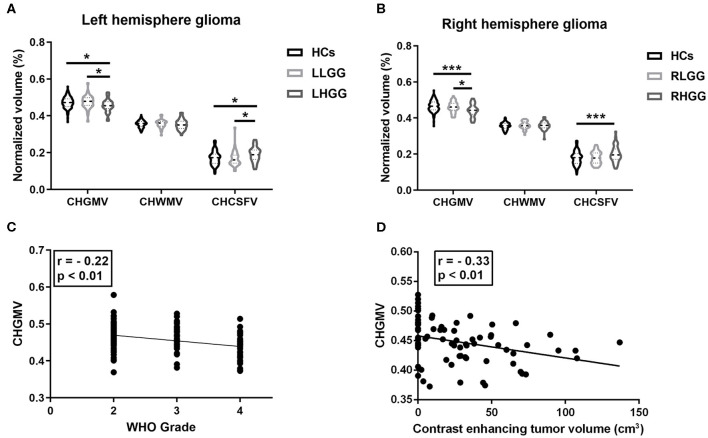
Brain structure alteration in the patients with gliomas. **(A)** In left hemisphere glioma, alterations of contra-hemispheric GM volume (CHGMV), WM volume (CHWMV), and CSF volume (CHCSFV) among LHGG group, LLGG group, and HCs (one-way ANOVA, Bonferroni corrected, *p < 0.05). **(B)** In right hemisphere glioma, alterations of CHGMV, CHWMV, and CHCSFV among RHGG group, RLGG group, and HCs (one-way ANOVA, Bonferroni corrected, *p < 0.05, ***p < 0.001). **(C)** Partial correlation analysis correcting for age between glioma grade and CHGMV in the patients. **(D)** In patients with high-grade gliomas, partial correlation analysis correcting for age between CHGMV and contrast enhanced volume.

In the left hemispheric gliomas, a one-way ANOVA of the LLGG, LHGG, and HCs was performed, and the main effect on the CHGMV (*F*_(2,188)_ = 4.37, *p* = 0.01) and CHCSFV (*F*_(2,188)_ = 4.11, *p* = 0.02) was significant. A Bonferroni's multiple comparisons test was performed, revealing a significantly decreased CHGMV (0.456 ± 0.034, 95% CI: 0.444–0.467) and increased CHCSFV (0.191 ± 0.040, 95% CI: 0.177–0.204) in the LHGG group compared to those in the HCs (CHGMV, 0.472 ± 0.031, 95% CI: 0.467–0.478; CHCSFV, 0.171 ± 0.037, 95% CI: 0.164–0.178) and LLGG group (CHGMV, 0.475 ± 0.036, 95% CI: 0.464–0.486; CHCSFV, 0.168 ± 0.042, 95% CI: 0.155–0.181) (Figure 2A, adjusted *p* < 0.05, CHGMV: LHGG vs. HCs, ES = 0.517, 95% CI: 0.1265–0.8838, LHGG vs. LLGG, ES = 0.5603, 95% CI: 0.1013–1.0193; CHCSFV: LHGG vs. HCs, ES = 0.4931, 95% CI: 0.1147–0.8715, LHGG vs. LLGG, ES = 0.5527, 95% CI: 0.094–1.0115). In the right hemisphere gliomas, a one-way ANOVA of the RLGG, RHGG, and HCs was performed, and the main effect on the CHGMV (*F*_(2,189)_ = 8.12, *p* = 0.0004) and CHCSFV (*F*_(2,189)_ = 4.34, *p* = 0.01) was significant. The results of the Bonferroni's multiple comparisons test showed CHGMV decreases in the RHGG group (0.442 ± 0.036, 95% CI: 0.431–0.453) compared to the HCs (0.465 ± 0.031, 95% CI: 0.460–0.471) and RLGG group (0.461 ± 0.030, 95% CI: 0.451–0.471) and CHCSFV increases in the RHGG group (0.200 ± 0.046, 95% CI: 0.185–0.214) compared to the HCs (0.179 ± 0.038, 95% CI: 0.172–0.186) (Figure 2B, adjusted *p* < 0.05, CHGMV: RHGG vs. HCs, ES = 0.6825, 95% CI: 0.3148–1.0502, RHGG vs. RLGG, ES = 0.5842, 95% CI: 0.1241–1.0369; CHCSFV: RHGG vs. HCs, ES = 0.4712, 95% CI: 0.1076–0.8348). There was no difference in the CHGMV, CHWMV, or CHCSFV between the HCs and low-grade glioma patients. Subsequently, we detected the relationship between the WHO grade and CHGMV in the glioma patients by performing a partial correlation analysis and correcting for age. As shown in Figure 2C, patients with a higher WHO grade showed a more significant decrease in the CHGMV (*r* = −0.22, *p* < 0.01). In the patients with high-grade glioma, the partial correlation analysis correcting for age revealed a significantly negative correlation between the CHGMV and contrast enhanced volume (Figure 2D, *r* = −0.33, *p* < 0.01).

### Predicting Tumor Molecular Markers in Patients With High-Grade Gliomas

In this section, age, the CHGMV, the lesion volume, and the contrast-enhanced volume were used to predict IDH1 mutations, P53 mutations, and ATRX loss. Both univariate and multivariate analyses were conducted to allow factors associated with the prediction of IDH1 mutations, P53 mutations, and ATRX loss to be identified. In the univariate analyses, the factors with statistical significance (*p* < 0.1) were selected for inclusion in the forward stepwise multivariate logistic regression analyses. The binary logistic regression modeling showed that the CHGMV was an independent predictor of IDH1 mutations (OR: 2.52E7, 95% CI: 6.361–1.00E14, *p* = 0.028, see Table 2) and P53 mutations (OR: 4.36E7, 95% CI: 10.753–1.77E14, *p* = 0.023, see Table 3). No significant factors predicting ATRX loss were detected.

**Table 2:** Univariate and multivariate logistic regression analysis of variables to predict IDH1 mutation.

**Variables**	**Univariate analysis**			**Multivariate analysis**		
	**B**	**OR (95% CI)**	***P*-value**	**B**	**OR (95% CI)**	***P*-value**
Age, year	−0.036	0.965 (0.926–1.004)	0.079	-	-	-
CHGMV	17.043	2.52E7 (6.361–1.00E14)	0.028	17.043	2.52E7 (6.361–1.00E14)	0.028
Lesion volume, cm^3^	−0.008	0.992 (0.982–1.002)	0.123	-	-	-
Contrast enhanced volume, cm^3^	−0.018	0.982 (0.965–0.999)	0.034	-	-	-
Constant	-	-	-	−7.216	-	0.036

**Table 3:** Univariate and multivariate logistic regression analysis of variables to predict P53 mutation.

**Variables**	**Univariate analysis**			**Multivariate analysis**		
	**B**	**OR (95% CI)**	***P*-value**	**B**	**OR (95% CI)**	***P*-value**
Age, year	−0.047	0.954 (0.915–0.994)	0.026	-	-	-
CHGMV	17.591	4.36E7 (10.753–1.77E14)	0.023	17.591	4.36E7 (10.753–1.77E14)	0.023
Lesion volume, cm^3^	0.511	1.667 (0.289–9.620)	0.568	-	-	-
Contrast enhanced volume, cm^3^	−0.012	0.988 (0.973–1.004)	0.150	-	-	-
Constant	-	-	-	−7.591	-	0.028

### Survival Predictions in Patients With High-Grade Glioma

**Figure 3:**
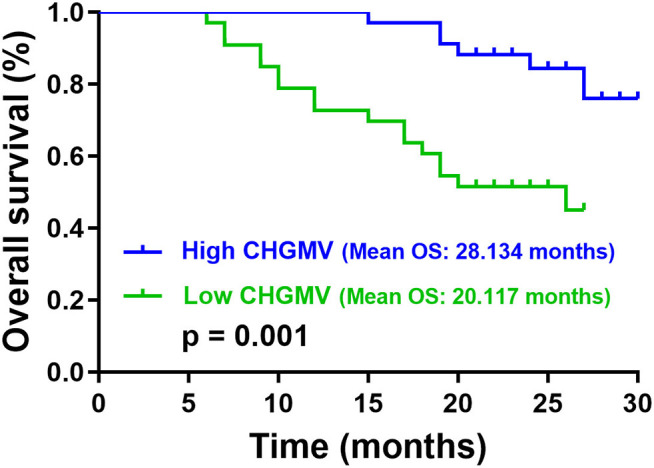
Overall survival (OS) analysis in patients with high-grade gliomas.

In the patients with high-grade gliomas, the median CHGMV was 0.446; thus, we classified the patients into a high CHGMV group (≥0.446, *n* = 34) and a low CHGMV group (<0.446, *n* = 33). Kaplan–Meier curves and log rank tests were used to evaluate the correlations between the CHGMV and OS, and the results suggested that a low CHGMV (mean OS: 20.117 months, 95% CI: 17.488–22.747 months) was associated with a shorter OS compared with a high CHGMV (mean OS: 28.134 months, 95% CI: 26.749–29.519 months) as shown in Figure 3 (*p* = 0.001). To further determine whether the CHGMV is an independent predictor of OS in high-grade glioma patients, we performed Cox proportional hazards regression analyses. First, a univariate Cox proportional hazards regression analysis was performed to evaluate the factors affecting OS in patients, such as the CHGMV, WHO grade (III and IV), age, contrast enhanced volume, KPS (classified into two subgroups, ≥80 and <80), IDH1 status, MGMT promoter methylation, P53 status, and ATRX expression (see Table 4). Second, all factors with a *p* < 0.1 were further evaluated by the forward stepwise multivariate Cox proportional hazards regression model, which suggested that a low CHGMV was an independent factor of poor OS (HR = 2.883, 95% CI: 1.075–7.735, *p* = 0.035) even after adjusting for the WHO grade (HR = 9.068, 95% CI: 2.629–31.282, *p* < 0.001), in the high-grade glioma patients (see Table 4).

**Table 4:** Univariate and multivariate Cox regression analysis of variables to predict OS.

**Variables**	**Univariate analysis**			**Multivariate analysis**		
	**B**	**HR (95% CI)**	***P*-value**	**B**	**HR (95% CI)**	***P*-value**
Age, year	0.045	1.046 (1.012–1.082)	0.007	-	-	-
CHGMV (High ≥ 0.446, Low < 0.446)	1.464	4.322 (1.684–11.088)	0.002	1.059	2.883 (1.075–7.735)	0.035
Contrast enhanced volume, cm^3^	0.012	1.012 (1.002–1.023)	0.016	-	-	-
KPS (≥ 80 and < 80)	1.228	3.414 (1.490–7.823)	0.004	-	-	-
histological grade (III/IV)	2.422	11.273 (3.332–38.142)	< 0.001	2.205	9.068 (2.629–31.282)	< 0.001
IDH1 (WT, M)	−1.489	0.226 (0.092–0.554)	0.001	-	-	-
ATRX (L, E)	0.989	2.688 (1.138–6.349)	0.024	-	-	-
P53 (WT, M)	−0.191	0.826 (0.364–1.875)	0.647	-	-	-
MGMT promoter methylation (U, M)	−0.439	0.644 (0.260–1.596)	0.342	-	-	-

*KPS, Karnofsky performance score; M, mutation; WT, wild type; E, expression; L, loss; U, unmethylated, M, methylated*.

## Discussion

High-grade gliomas are the most common primary malignant intracranial tumors and have a high mortality rate. In gliomas, to avoid the influence of tumor occupation, the contrahemispheric brain has been used to investigate the effects of chemoradiation on the brain structure in a previous study (28). Furthermore, contralesional local brain regions, including the insula and hippocampus, have been studied in glioma (29, 30). To date, whether a unilateral glioma affects the contralesional hemisphere structure has not been studied. In the present study, we first investigated the different effects of low- and high-grade gliomas on the contralesional hemisphere structure. Our results demonstrate atrophy of contrahemispheric cortex in high-grade glioma patients. More importantly, we found that the contrahemispheric gray matter volume (CHGMV) can independently predict tumor molecular markers and OS in patients with high-grade glioma.

Traditionally, the roles of glioma in brain tissue, including infiltration and destruction, are believed to be only local. In recent years, immunohistochemical data using antibodies specific to IDH1 R132H mutant protein have revealed a more widespread tumor cell distribution than expected, including in the remote contrahemisphere, and have redefined high-grade glioma as a whole-brain disease (20). Notably, our study indicated that unilateral high-grade glioma not only affected the surrounding brain tissue, but also led to atrophy of contrahemispheric cortex for the first time with 3-D T1 imaging. Moreover, the correlation analysis showed that a lower CHGMV is associated with a higher glioma grade. These results strongly support the previously believed concept that glioma is a whole-brain disease. In addition, our results showed a significant correlation between a lower CHGMV and a higher contrast enhanced volume in high-grade gliomas. Multiple studies have confirmed that areas of contrast enhancement on MRI consistently contain the highest density of tumor cells along with the most aggressive histological features in malignant glioma (31–33). The decrease in CHGMV may be secondary to the fast progression of tumor cells. In addition, abnormal amounts of neurotransmitter release from glioma cells and inflammation caused by tumor may also contribute to this finding. Abnormal concentrations of glutamate, which is released from glioma cells, have been reported to lead to widespread neuronal death *in vitro* (34). Inflammation has been shown to be detrimental to normal brain tissue in animal study and tumor-associated macrophages/microglia have been demonstrated to accumulate in glioma (35, 36). No atrophy of the contrahemispheric cortex was found in the unilateral low-grade glioma patients compare to HCs, possibly due to compensatory alterations, which appear to be substantially more effective in cases of progressive rather than acute injury in contrahemispheric cortex (37). Similarly, Herbet et al. reported compensatory alterations in the contralesional cortex in diffuse low-grade glioma (30).

Previously, clinical variables, such as a lower age, high KPS, lower histological grade, less infiltrating tumor, lower volumes of enhancing tumor, and lower edema volume, have been associated with a longer OS in patients with high-grade glioma (18, 38–40). In glioma, tumor molecular markers, such as IDH1, P53, MGMT promoter methylation, and ATRX, are important prognostic factors for therapy response and OS (41–43). Previous studies have suggested that IDH1 mutations, P53 mutations, MGMT promoter methylation, and ATRX loss indicated a good therapy response and longer OS in high-grade glioma (41, 43, 44). In view of the extent of the tumor burden on the contrahemispheric cortex might offer prognostically valuable information, our study examined the relationship between the CHGMV and OS in high-grade glioma patients with information regarding OS and pathological indexes. Interestingly, our results showed that patients with high CHGMV experience longer OS than those with low CHGMV. Furthermore, Cox proportional hazards regression analysis was performed to investigate the predictive value of the CHGMV. Considering that the WHO grade (III and IV), age, KPS, contrast-enhanced volume, IDH1 status, P53 status, MGMT promoter methylation, and ATRX expression are associated with OS in patients with high-grade glioma, these factors were also analyzed in Cox proportional hazards regression analysis. Remarkably, the result showed that low CHGMV was an independent factor for worse OS.

In radiogenomics, multiple imaging features, including structural, hemodynamic, and physiological information, are used to predict molecular markers before surgery and found MRI features may also serve as non-invasive biomarkers of underlying molecular events (45–48). In addition to predicting OS, in our study, the CHGMV was also used to predict IDH1 mutation, P53 mutation, and ATRX loss using binary logistic regression analysis in high-grade glioma. The results demonstrated that the CHGMV can independently predict IDH1 and P53 mutation. High CHGMV suggested an increased possibility of IDH and P53 mutations in high-grade gliomas. Thus, the CHGMV can be used as a non-invasive biomarker to predict molecular markers before surgery.

There are several limitations to our study. First, the assessment of the CHGMV can only be performed in patients with no shift of the midline due to tumor space occupation and bilateral extension of the lesion. Second, our cohort was small in size and from a single institution. Larger data from other institutions are needed to confirm and validate the reported findings. Third, not all patients collected the entire pathological indexes (IDH1, ATRX, MGMT, and p53). Another limitation of our study is that the time of follow-up was short, and half of the patients did not reach the end event.

In conclusion, we found contrahemispheric cortical atrophy in unilateral high-grade glioma and that the CHGMV is significantly correlated with the glioma grade and contrast-enhanced volume for the first time. These results strongly support the previously believed concept that glioma is a whole-brain disease. More importantly, the CHGMV can be used as a promising imaging biomarker to predict the OS and molecular markers in patients with high-grade gliomas. The CHGMV has great prospects in clinical applications for prognostic prediction and guidance of diagnosis and treatment of high-grade glioma patients.

## Data Availability Statement

The datasets generated for this study are available on request to the corresponding author.

## Ethics Statement

The studies involving human participants were reviewed and approved by the Medical Ethics Committee of Beijing Tiantan Hospital. The patients/participants provided their written informed consent to participate in this study.

## Author Contributions

TY, CL, and YZ: designed and conceptualized study. TY, JY, LJ, CL, SG, GL, and ZG: major role in the acquisition of data. TY, ZZ, JY, and RW: analyzed the data. TY: drafted the manuscript for intellectual content. CL and YZ: revised the manuscript for intellectual content. All authors: contributed to the article and approved the submitted version.

## Conflict of Interest

The authors declare that the research was conducted in the absence of any commercial or financial relationships that could be construed as a potential conflict of interest.
