# Supplementary material for: Contrahemispheric Cortex Predicts Survival and Molecular Markers in Patients With Unilateral High-Grade Gliomas
Source: Front Oncol. 2020 Jul 23;10:953. doi: 10.3389/fonc.2020.00953 (PMC7390929; doi:10.3389/fonc.2020.00953)
Supplement: Supplementary file 1 [file Table_1.DOCX]

Supplementary Table 1. Tumor types and tumor locations.

| Variable | LLGG  group | LHGG  group | RLGG group | RHGG  group | P value |
| --- | --- | --- | --- | --- | --- |
| **Tumor types** |  |  |  |  |  |
| Astrocytoma/ Oligodendrogliom | 16/24 | NA | 13/24 | NA | P=0.66^a^ |
| Anaplastic glioma/ glioblastoma | NA | 21/15 | NA | 15/25 | P=0.07^a^ |
| **Tumor locations** |  |  |  |  |  |
| Frontal/ Temporal/Frontal and temporal/ Parietal/ Occipital/Thalamus | 20/12/5/2/1/0 | 19/8/6/1/1/1 | 22/6/8/1/0/0 | 16/9/5/4/3/3 | P=0.36^a^ |

^a^ represent χ2 test.
